# Association of Age and Hospitalization Amongst Those with Underlying High-risk Conditions at COVID-19 Diagnosis in a Large, State-wide Health System

**DOI:** 10.1007/s11606-021-06942-y

**Published:** 2021-06-16

**Authors:** Jonathan H. Watanabe, Jimmy Kwon, Sanjay R. Mehta

**Affiliations:** 1 Department of Clinical Pharmacy Practice, School of Pharmacy & Pharmaceutical Sciences, University of California Irvine, Irvine, CA USA; 2 Department of Statistics, Donald Bren School of Information and Computers Sciences, University of California Irvine, Irvine, CA USA; 3 Department of Medicine, School of Medicine, University of California San Diego, La Jolla, CA USA

## BACKGROUND

Prior studies have demonstrated increased mortality for older adults and patients with high-risk underlying conditions and COVID-19.^1^ However, the additional likelihood of hospitalization for older adults with these conditions compared to younger patients is unknown.

## OBJECTIVE

The objective of this study was to measure the increased odds of hospitalization for those 65 to 74 years old and >75 years old compared to those <65 years old with high-risk conditions at COVID-19 diagnosis.

## METHODS

Data for 25,834 patients with SARS-CoV-2 infection, diagnosed by RNA nucleic acid amplification, were analyzed for March 10, 2020, through January 14, 2021, from the University of California COVID Research Data Set (UC CORDS). UC CORDS contains SARS-CoV-2 testing results and COVID-19 treatment information collected from across University of California (UC) Health and is distributed weekly for research purposes. Per UC Health reporting, patients were designated as having a COVID-19-related hospitalization if admitted within 30-days of diagnosis or tested positive during the stay. Presence of high-risk conditions hypertension, hyperlipidemia, heart disease, type 2 diabetes, cancer, asthma/chronic obstructive pulmonary disorder (COPD), chronic kidney disease (CKD), and end-stage renal disease (ESRD) was determined using the *International Classification of Diseases, Tenth Revision, Clinical Modification* (ICD-10-CM) codes in the electronic health record (EHR). A combined cardiovascular risk factors category that included patients with hypertension, hyperlipidemia, or heart disease was also assessed. Preexisting conditions included ICD-10-CM codes up to one-year preceding COVID-19 diagnosis. Multiple logistic regression was used for estimation of odds ratios. Exposure variables were older adult categories defined as 65 to 74 years old and >75 years old. Outcome variable was COVID-19-related hospitalization. Adjustment variables were gender and race/ethnicity extracted from EHR. All analyses performed in R, version 3.6.3 (R Project for Statistical Computing). Statistical significance set at level of alpha = 0.05. UC CORDS was operationalized by UC Health as “non-human subjects research” and analyses are considered institutional review board exempt.

## FINDINGS

**Figure 1:**
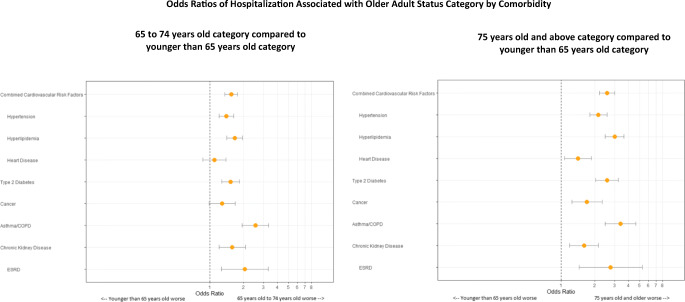
Odds Ratios of Hospitalization Associated with Older Adult Status Category by Comorbidity.

The mean [SD] age of the study population was 42.4 [20.6] years. The sample was 53% female, 43% were White, 22% non-White Hispanic, 7% Asian, 6% Black, and 44% reported as “Other” (Table 1). For each high-risk condition group, those 65 to 74 years old and >75 were at increased odds of hospitalization compared to patients < 65. All differences between older adult categories and <65 reference category were statistically significant except for heart disease and cancer in the 65- to 74-year-old category. The largest OR was 3.38 [95% CI, 2.47 to 4.62] for patients >75 with asthma/COPD. The magnitude in odds of hospitalization for those >75 increased to an OR of 2.76 [95% CI, 1.44 to 5.28] for the ESRD group compared to OR of 1.60 [95% CI, 1.19 to 2.14] for the CKD group. The OR for patients with either hypertension, hyperlipidemia, or heart disease was 1.55 [95% CI, 1.36 to 1.77] in 65- to 74-year-olds and 2.56 [95% CI, 2.19 to 3.01] in those >75 (Fig. 1).

**Table 1:** Patient Characteristics

Total number of patients	25,834 (100%)
Hospitalized patients	4146 (16%)
Gender	
Female	13,667 (53%)
Race and ethnicity	
Non-White Hispanic	5668 (22%)
White	11,044 (43%)
Black	1482 (6%)
Asian	1859 (7%)
Other	11,449 (44%)
Overall mean age	42.4 ± 20.5
Under 65 years old age group	21,667 (84%)
Under 65 years old age group mean	36.7 ± 16.2
Between 65 and 74 years old age group	2352 (9%)
Between 65 and 74 years old age group mean	69.0 ± 2.8
75 years and older age group	1815 (7%)
75 years and older age group mean	81.8 ± 4.6
Conditions	
Combined cardiovascular risk factors	6602 (26%)
Hypertension	4733 (18%)
Hyperlipidemia	4219 (16%)
Heart disease	1667 (6%)
Type 2 diabetes	2972 (12%)
Cancer	1619 (6%)
Asthma/COPD	1973 (8%)
Chronic kidney disease	1504 (6%)
ESRD	562 (2%)

## DISCUSSION

In this analysis of COVID-19-positive patients in a large, diverse, state-wide health system, adults >65 years with underlying high-risk conditions were much more likely to be hospitalized following SARS-CoV-2 infection than younger patients with high-risk conditions adjusted for gender and race/ethnicity. The observational nature of this study precludes confirmation of a cause and effect relationship between age and hospitalization, but rather demonstrated an association between older age and hospitalization. As of April 15, 2021, COVID-19 vaccination is available to all adults in the USA.^2^ However, a significant portion of the highest risk population (adults >65 years old and adults with high-risk conditions) remain unvaccinated accompanied by a troubling slowing rate of vaccination in older adults.^3^ As cases and hospitalizations persist^4^ and highly transmissible SARS-CoV-2 variants gain dominance,^5^ outreach efforts to vaccinate these high-risk unvaccinated individuals is critical. We will need to rethink our vaccination strategies to significantly improve convenience and outreach to reach this population. Full vaccination (i.e., 2 doses of the mRNA-based vaccines) of this population is crucial to ensure this most vulnerable population is protected as rapidly as possible. Furthermore, in the high-risk individuals that become infected with SARS-CoV-2, we should prioritize monoclonal antibody therapies, which have shown a benefit in these persons.^6^

## Declarations

### Disclaimer

The views expressed in this manuscript do not represent the official views of the National Academies of Sciences, Engineering, and Medicine.
